# The Utility of Fourier Transform Infrared Spectroscopy (FTIR) for Detecting Exercise‐Induced Changes in the Human Hand Epidermis

**DOI:** 10.1002/jbio.202500173

**Published:** 2025-06-22

**Authors:** Paweł Król, Zbigniew Obmiński, Adam Reich, Wojciech Czarny, Józef Cebulski, Joanna Depciuch, Michał Zamorski, Katarzyna Stępień, Łukasz Rydzik

**Affiliations:** ^1^ Department of Physical Education Medical College of Rzeszow University Rzeszow Poland; ^2^ Department of Endocrinology Institute of Sport—National Research Institute Warsaw Poland; ^3^ Department of Dermatology Medical College of Rzeszow University Rzeszow Poland; ^4^ Institute of Physics, Faculty of Exact and Technical Sciences University of Rzeszow Rzeszow Poland; ^5^ Institute of Nuclear Physics, Polish Academy of Sciences Kraków Poland; ^6^ Department of Biochemistry and Molecular Biology Medical University of Lublin Lublin Poland; ^7^ Department of Sport Theory and Motor Skills, Institute of Sport Sciences University of Physical Culture in Kraków Kraków Poland

**Keywords:** absorbance, biochemical changes, epidermis, Fourier transform infrared spectroscopy (FTIR), physical exercise

## Abstract

The literature lacks data on transient infrared spectral changes in the epidermis following physical exercise. This study tested the hypothesis that a single exercise session affects selected spectral bands (3270–1045 cm^−1^) in healthy individuals. Eight professional tennis players completed a 1.5‐h moderate‐intensity training session. Epidermal samples from the inner hand were collected before and after exercise, following cleaning with distilled water and 96% PA ethyl alcohol. Samples were analyzed using Fourier Transform Infrared Spectroscopy (FTIR). Absorbance values were recorded for 12 peaks. Significant correlations were observed for the 3270 cm^−1^ (*r* = 0.976) and 1045 cm^−1^ (*r* = 0.754) peaks. Notably, post‐exercise increases were found at 1453 cm^−1^ (lipids/proteins), 1078 cm^−1^ (phospholipids), and 1045 cm^−1^ (carbohydrates). No significant changes were observed for other peaks, though a general upward trend appeared. Inter‐individual variability was high. FTIR may detect acute epidermal biochemical responses to exercise, especially in lipid‐ and phospholipid‐related structures.

## Graphical abstract

This study demonstrates that physical exercise induces measurable biochemical changes in the epidermis. FTIR spectroscopy detected significant post‐exercise increases in spectral peaks related to lipids, phospholipids, and carbohydrates, highlighting the method's potential for non‐invasive skin and metabolism monitoring in athletes.

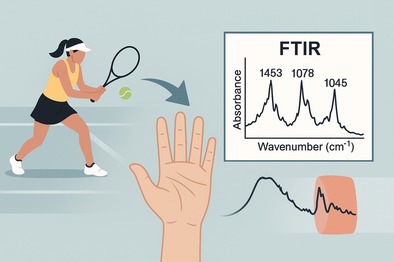

## 1 Introduction

Fourier Transform Infrared Spectroscopy (FTIR) is a method used in clinical research for diagnosing morphological changes in biological tissues. Specific wavenumber values are assigned to various chemical bonds in organic molecules. The first results were presented in the early 1990s [1]. Since then, FTIR spectroscopy has been continuously improved, with advancements in the identification of chemical bonds and a broad range of applications in medical diagnostics [2, 3, 4] and forensic science [5, 6].

FTIR spectroscopy has proven useful in assessing the physiological state of the skin and epidermis. It allows for the identification of various lipids and the secondary struc‐ture of proteins present in skin fibroblasts and epidermal keratinocytes. Infrared spectral analysis has shown a highly similar spectral shape between living skin, living epidermis, and cultivated substitutes, with the only differences being in absorbance values [7]. FTIR has also been found useful in detecting very early changes characteristic of developing cancer in various tissues, including the skin [8, 9, 10]. The spectra of healthy and can‐cer‐threatened tissues differ in the so‐called “diagnostic spectral regions”: 3300–2850 cm^−1^, 1700–1500 cm^−1^, and 850–800 cm^−1^. Similar changes in infrared spectra have been reported in skin damaged by electric current flow [11, 12].

Notably, this technique has demonstrated that, in healthy individuals, infrared spectral differences depend on age. Skin aging markers include glycosaminoglycans and protein functional groups. In older individuals, a decrease in absorbance by several tens of percent has been observed for peaks in several infrared radiation bands: 1259–1223 cm^−1^, 1738–1646 cm^−1^, 1636–1523 cm^−1^, 1511–1457 cm^−1^, and 1218–1139 cm^−1^ [13]. Another study on skin revealed an age‐related decline in absorbance characteristic of amides and collagen [14]. It has been shown that these changes can be mitigated by regular aerobic exercise, which results in a reduction in the thickness of the stratum corneum and an increase in collagen content in the skin [15]. Recently, FTIR has been used to detect spectral changes in fibroblasts in individuals developing multiple sclerosis [16].

A particularly important research area involving FTIR is the physiology and bio‐chemistry of epidermal cells, as the epidermis serves as the first barrier protecting the body from external environmental factors, including thermal, chemical, and biological pathogens [17, 18]. A key component of the physiological barrier against microorganisms and infections is secretory immunoglobulin A (SIgA) found in sweat. Intense exercise af‐fects the immune system, leading to a significant decrease in SIgA levels and an increase in β‐defensin 2 (HBD‐2) [19].

Biochemical processes, including the synthesis and metabolism of biologically active steroid hormones such as DHEA‐S, DHEA, and 17β‐estradiol, occur in living keratinocytes, stimulating the migration of damaged keratinocytes [20, 21]. Keratinocytes also synthesize lipids such as ceramides, cholesterol, and fatty acids [22], although these lipids can also enter the epidermis from circulation via transporting glycoproteins. Consequently, ceramides, cholesterol, and fatty acids within keratinocytes collectively constitute 100% of all lipid content [23], playing a crucial role in maintaining proper transport of biologically active substances across cell membranes and regulating intracellular metabolism [24]. The rate of these processes may change depending on the season. Seasonal variations in absorbance have been observed for certain compounds, such as hydroxy acids [25]. Interestingly, the authors of this study also reported differences in epidermal absorbance at different anatomical sites, including the arms, fingers, neck, and nose, as well as interindividual differences in absorbance, expressed as a coefficient of variation (CV%) ranging from 15% to 20%.

Despite extensive research, the available literature lacks information regarding changes in the infrared spectrum of the epidermis following a single bout of physical exercise. The aim of this study was to analyze changes in the infrared spectrum of the epidermis after a 1.5‐h moderate‐intensity training session.

## 2 Materials and Methods

### 2.1 Participants

The study was conducted on a group of eight male professional tennis players aged 22 to 26 years (exact age ranges to be specified). All participants were healthy and free from dermatological or systemic conditions that could influence the study results. Before participating in the experiment, each subject provided informed consent, and the study protocol was approved by the Bioethics Committee at the University of Rzeszów (Resolution No. 8/11/2020). All procedures adhered to the principles of the Declaration of Helsinki. The average age of participants was 24.1 ± 1.6 years. All athletes had a minimum of 8 years of professional training experience and participated in regular national‐level competitions. Hydration status was not directly measured; however, participants were instructed to maintain usual hydration prior to testing, and no fluid intake restrictions were applied before the session.

### 2.2 Study Design

The study followed a quasi‐experimental repeated‐measures (pre‐post) design, in which biochemical variables of the epidermis were assessed before and after physical exercise. The training session lasted 1.5 h and was of moderate intensity, tailored to the individual fitness levels of the participants. The training took place on a tennis court, and exercise intensity was monitored using heart rate indicators and the subjective scale of perceived exertion (SPECTRUM). The training was conducted in the morning in a professional indoor facility equipped with ventilation and air conditioning, maintaining a temperature of 20°C–22°C.

### 2.3 Epidermal Sample Collection

Epidermal samples were collected from the inner side of the hand (palmar surface, central area). Before training and sample collection, the skin surface was cleaned by washing the sampling site with distilled water to remove hydrophilic contaminants, followed by 96% ethanol to eliminate hydrophobic impurities. This preparation aimed to minimize the influence of sweat components and sebum accumulated on the stratum corneum. Each sample was collected under standardized hygienic conditions using sterile tools, and the epidermis was obtained through a gentle stratification technique.

### 2.4 Instrumentation and Analysis Conditions

The analysis was performed using a Bruker Alpha II FTIR spectrometer equipped with an ATR (Attenuated Total Reflection) module. The spectral resolution was set at 2 cm^−1^, and the spectrometer was calibrated using standard polystyrene film before each measurement session in accordance with manufacturer recommendations. The spectrometer operated in the Attenuated Total Reflection (ATR) mode, allowing direct sample analysis. Spectra were recorded in the range of 3270 cm^−1^ to 1045 cm^−1^ with a spectral resolution of 2 cm^−1^. Each sample was measured multiple times (at least three measurements per sample) to minimize measurement errors and obtain mean absorbance values.

### 2.5 Spectral Interpretation

The data analysis focused on the absorbance values of 12 characteristic spectral bands corresponding to specific chemical bonds in biochemical components of the epidermis. The following bands were interpreted: 3270 cm^−1^ (O—H bonds in water), 2956, 2922, and 2852 cm^−1^ (CH_3_, CH_2_ bonds in lipids, phospholipids, ceramides, and fatty acids), 1736 and 1642 cm^−1^ (C=O bonds in phospholipids/lipids and primary amides), 1453 cm^−1^ (CH_2_ and CH_3_ bonds in proteins and lipids), and 1078 and 1045 cm^−1^ (phospholipids and carbohydrates). All spectral interpretations were based on existing FTIR spectroscopy literature for biological tissues (Table 1).

**TABLE 1:** Spectral Interpretation with FTIR.

Band position	Assignment	Structure
3270 cm^−1^	vs O—H	Water
2956 cm^−1^	vas CH_3_	Proteins phospholipids, ceramides, fatty acids
2922 cm^−1^	vas CH_2_	Lipids, phospholipids, ceramides, fatty acids
2852 cm^−1^	vas CH_2_	Lipids, phospholipids, ceramides, fatty acids
1736 cm^−1^	v C=O	Phospholipids, esters, glycerides
1642 cm^−1^	v C=O	Amide I, C=O stretch
1536 cm^−1^	δ(N‐H), v CN	Amide II, C—N stretch
1453 cm^−1^	δ(CH _2_), δas (CH_3_)	Proteins, lipids
1393 cm^−1^	δs (CH_3_)	Amino acid chains in peptides and proteins
1242 cm^−1^	v (CN), δ(NH), vas(PO_2_^−^)	Amide III, C—N stretch, N—H, Methyl‐C stretch
1078 cm^−1^	vs (PO_2_^−^)	Phospholipids
1045 cm^−1^	v (CO), δ(CO)	C—OH carbohydrates

### 2.6 Statistical Analysis

All data were subjected to statistical analysis using STATISTICA software (version 13.3). Due to the lack of normal data distribution, verified by the Shapiro–Wilk test, the non‐parametric Wilcoxon signed‐rank test was used to compare absorbance values before and after training. Relationships between selected variables were assessed using Spearman's correlation coefficients. Statistical significance was set at *p* < 0.05. The results are presented as mean absorbance values with standard deviations and coefficients of variation (CV%). All calculations were thoroughly verified and repeated to ensure accuracy.

## 3 Results

Absorbance values and statistical calculation results are presented in Tables 2 and 3.

**TABLE 2:** Mean absorbance values, standard deviations, and coefficients of variation for peaks in the infrared spectrum range from 3270 to 1642 cm^−1^.

Scores of absorbance of infrared spectrum expressed for peaks of various wavenumber*cm^−1^							
Sampling	Variable	3270	2956	2922	2822	1736	1642
Pre training	X	1.276	0.739	1.057	0.386	0.166	1.859
	SD	0.256	0.219	0.382	0.069	0.115	0.265
	CV%	20.1	29.6	36.1	17.9	69.3	14.3
	Min	1.061	0.553	0.708	0.282	0.088	1.338
	Max	1.489	1.181	1.945	0.474	0.462	2.000
Post training	X	1.348	0.787	1.117	0.447	0.200	1.939
	SD	0.322	0.247	0.288	0.127	0.084	0.068
	CV%	23.9	31.4	25.8	28.4	42.0	3.5
	Min	1.080	0.647	0.705	0.214	0.069	1.842
	Max	1.618	1.223	1.575	0.585	0.312	1.999
Rho		**0.976**	0.381	0.611	0.476	0.690	0.166
*Z*‐value		1.540	0.840	0.420	1.400	1.120	0.420
*p*‐value		0.123	0.401	0.674	0.161	0.263	0.674
Post‐pre diff.		+5.6%	+6.5	+5.7	+15.8	+20.5	+4.3

**TABLE 3:** Mean absorbance values, standard deviations, and coefficients of variation for peaks in the infrared spectrum range from 1536 to 1045 cm^−1^.

Scores of absorbance of infrared spectrum expressed for peaks of various wavenumber*cm^−1^							
Sampling	Variable	1536	1453	1393	1242	1078	1045
Pre training	X	1.586	0.799	0.806	0.762	0.479	0.597
	SD	0.171	0.112	0.118	0.089	0.204	0.303
	CV%	10.8	15.3	14.6	11.7	42.6	50.8
	Min	1.269	0.575	0.656	0.617	0.223	0.225
	Max	1.745	0.909	0.949	0.917	0.782	1.127
Post training	X	1.635	0.960	0.941	0.773	0.812	0.879
	SD	0.189	0.155	0.197	0.179	0.363	0.398
	CV%	11.6	16.1	20.9	23.2	44.7	45.3
	Min	1.322	0.634	0.578	0.473	0.278	0.290
	Max	1.822	1.138	1.227	0.989	1.311	1.345
Rho		−0.500	0.096	0.048	−0.143	0.238	**0.754**
*Z*‐value		0.560	**2.100**	1.540	0.00	**2.380**	**2.521**
*p*‐value		0.575	**0.036**	0.123	1.00	**0.017**	**0.012**
Post‐pre diff.		+3.1%	+20.2%	+16.8%	+1.1%	+69.5%	+47.2%

**FIGURE 1:**
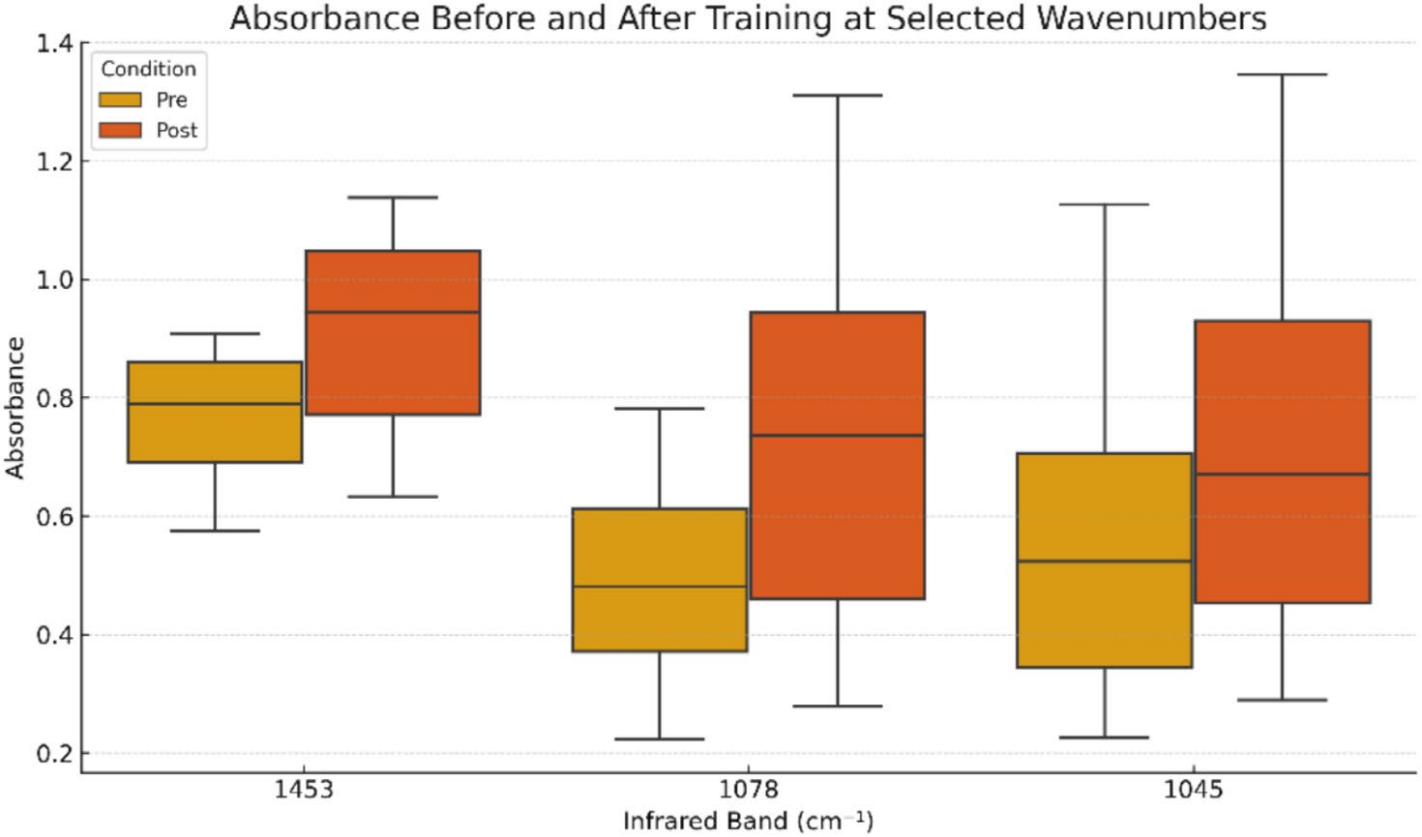
Boxplot representation of absorbance values at selected wavenumbers (1453, 1078, and 1045 cm^−1^) before and after physical training. Each box shows the distribution across eight participants. Post‐exercise increases in absorbance were significant for all three spectral bands.

A graphical comparison of absorbance values before and after exercise at wavenumbers 1453, 1078, and 1045 cm^−1^ is presented in Figure 1. These spectral bands showed statistically significant increases and are associated with proteins, lipids, phospholipids, and carbohydrates.

The study results showed a statistically significant post‐exercise increase in absorbance for infrared radiation peaks at wavenumbers 1453, 1078, and 1045 cm^−1^. According to the interpretation key provided in Table 1, these values correspond to the bands (δ CH_2_), δas CH_3_ for proteins and lipids, versus PO_2_^−^ for phospholipids, and v CO, and δCO for carbohydrates. For the absorbance of the remaining peaks, post‐exercise changes were not statistically significant but showed a slight upward trend. Interindividual relative differences in absorbance values for various peaks, expressed as coefficients of variation (CV%), were observed both before and after exercise.

The data displayed in Tables 2 and 3 reveal considerable interindividual variability in absorbance for bands with different wavenumbers. The magnitude of this variability corresponds to the high values of the coefficients of variation (CV%). Heart rate (145–158 bpm) and perceived exertion levels (68%–71%) confirmed moderate exercise intensity consistent with match‐like conditions.

## 4 Discussion

A single bout of physical exercise induces temporary biochemical changes in the blood. Short but highly intense exercise leads to an increase in glucose and lactate concentrations [26, 27]. In contrast, low‐intensity but prolonged exercise results in increased concentrations of fatty acids and glycerol while reducing triglyceride levels [28]. The biochemical response to a tennis match is somewhat similar to that observed after prolonged exercise. During a two‐hour match, a monotonic increase in glycerol and free fatty acid concentrations was observed, while glucose levels remained stable and lactate concentrations were relatively low [29].

In our study, biochemical responses were not assessed, and exercise intensity evaluation was based on several heart rate measurements taken after more intense actions, as is typical during interval training. The results are comparable to HR values recorded in professional tennis players during a one‐hour simulated match [30].

As previously, mentioned, fatty acids penetrate from the blood into the epidermis through transport proteins. This suggests that the increase in their content in the epidermis could be partially attributed to post‐exercise changes in the concentration of these metabolites in the blood.

The increased absorbance intensity for selected peaks (1453, 1078, and 1045 cm^−1^) indicates quantitative changes in biochemical components of the epidermis, such as carbohydrates, proteins, and lipids. Significant correlation coefficients between pre‐ and post‐exercise data suggest parallel and quantitatively similar post‐exercise increases in absorbance. The contribution of molecules contained in sweat to post‐exercise changes in the epidermal spectrum is important but difficult to estimate. Sweat may contribute to the observed spectral changes, as it contains various metabolites such as lactate, glucose, and urea [31, 32, 33, 34, 35]. The characteristic wavenumbers for urea are 3461 and 1641 cm^−1^ [36]. The dominant peak at 3461 cm^−1^ falls outside the measurement range of our study, while the second band showed a slight mean post‐exercise increase in absorbance and significant interindividual differences before training. The interpretation of urea's contribution to this band is complicated by urea‐water interactions and the concentration‐dependent aggregation of this compound [37]. The presence of lipids in sweat suggests that this could be a transport mechanism for these compounds to the epidermis [38, 39, 40].

A critical methodological requirement is the pre‐analytical procedure. In our study, this included cleaning the stratum corneum with water and an organic solvent before training and sample collection. Other researchers have used chloroform and isopropanol [41] or water with soap [25] for this purpose. The removal of natural endogenous lipids from the skin surface before sampling significantly reduces the ratio of lipid absorbance at 2920 cm^−1^ to water absorbance at 3300 cm^−1^ in vivo measurements [41].

In our study, interindividual variability in absorbance before and after exercise was similar for each of the 12 peaks, except for the high variability observed in the two longest infrared wavelengths corresponding to 1078 and 1045 cm^−1^. Additionally, a significant correlation coefficient was recorded between pre‐ and post‐exercise absorbance for the 3270 cm^−1^ peak, which corresponds to O—H water bonds. A slight post‐exercise increase in absorbance could result from increased epidermal hydration. The level of epidermal hydration may be influenced by sweat secretion, which depends on factors such as body composition, environmental conditions, and fluid intake, as demonstrated in soccer players during a single training session in elevated temperature and humidity conditions [42].

A significant post‐exercise increase in absorbance at 1045 cm^−1^, associated with carbohydrates, may indicate an increased presence of glucose and glycerol in epidermal samples after exercise. The increase in absorbance at 1078 and 1453 cm^−1^ suggests a higher content of phospholipids and lipids in epidermal samples post‐exercise. Clarifying the extent of metabolite transport from circulation to the epidermis highlights the need to integrate FTIR epidermal analysis with FTIR‐based measurements of metabolite concentrations in blood and sweat, which is now possible due to the ongoing development of advanced analytical techniques [43, 44, 45, 46, 47].

### 4.1 Study Limitations

This study has several limitations that should be considered when interpreting the results. It was conducted on a small group of eight tennis players, which limits the generalizability of the findings to a broader population and different types of physical activity. The lack of simultaneous biochemical measurements in blood and sweat makes it difficult to fully understand the mechanisms of metabolite transport between blood, skin, and epidermis. Additionally, the study focused on a single exercise session, preventing any conclusions about long‐term biochemical changes.

## 5 Conclusions

1. A statistically significant increase in absorbance was observed for bands associated with lipids, phospholipids, and carbohydrates. A particularly significant increase was noted for the peaks at 1453 cm^−1^ (lipids), 1078 cm^−1^ (phospholipids), and 1045 cm^−1^ (carbohydrates), suggesting an increased presence of these compounds in the epidermis after exercise.
2. The results revealed substantial interindividual differences both before and after training, indicating individual biochemical variability in response to physical exercise.
3. A slight increase in absorbance at 3270 cm^−1^, attributed to O—H water bonds, may indicate an increase in water content in the epidermis due to exercise, likely resulting from enhanced sweat secretion.
4. For most of the other analyzed absorbance peaks, no significant changes were observed post‐training, although they exhibited a general upward trend.
5. The variability within samples and interindividual differences requires further research to better understand the mechanisms responsible for the observed changes.

## 6 Practical Applications

1. FTIR spectroscopy could be applied in monitoring short‐term biochemical changes in the epidermis related to physical activity. The findings suggest that FTIR may serve as a tool for assessing metabolism and skin condition post‐exercise, which could be useful in sports, dermatology, and esthetic medicine.
2. Given the substantial interindividual differences in epidermal response to exercise, this technique could be employed to personalize training and therapeutic approaches, adapting them to the individual needs of patients.
3. The study results may contribute to the development of portable devices for real‐time monitoring of biochemical changes in the epidermis, which could support diagnostics and therapy in the context of skin protection and athlete health.

## Author Contributions

Conceptualization, Paweł Król and Y.Y.; methodology, Paweł Król, Zbigniew Obmiński; software, Paweł Król, Joanna Depciuch, Michał Zamorski; validation, Paweł Król, Michał Zamorski and Wojciech Czarny; formal analysis, Paweł Król, Łukasz Rydzik, Józef Cebulski, Adam Reich; investigation, Paweł Król; resources, Paweł Król, Adam Reich, Józef Cebulski, Katarzyna Stępień; data curation, Paweł Król, Katarzyna Stępień; writing – original draft preparation, Paweł Król, Zbigniew Obmiński; writing – review and editing, Paweł Król, Łukasz Rydzik, Józef Cebulski, Adam Reich, Zbigniew Obmiński; visualization, Paweł Król, Joanna Depciuch, Wojciech Czarny; supervision, Paweł Król, Łukasz Rydzik; project administration, Paweł Król; funding acquisition, Paweł Król, Adam Reich, Wojciech Czarny All authors have read and agreed to the published version of the manuscript.

## Ethics Statement

The study was conducted in accordance with the Declaration of Helsinki, and approved by the Ethics Committee of University of Rzeszow (protocol code 8/11/2020 and date of 8.12.2020).

## Consent

Informed consent was obtained from all subjects involved in the study.

## Conflicts of Interest

The authors declare no conflicts of interest.

## Data Availability

Data sharing is not applicable to this article as no new data were created or analyzed in this study.
